# Early-Closing Day at Chemists' Shops

**Published:** 1914-11-21

**Authors:** Albert Larking

**Affiliations:** Secretary. Early Closing Association, 3 Tudor Street, E.C.


### THE EDITOR'S LETTER-BOX.

### Early-closing Day at Chemists' Shops.

*To the Editor of* The Hospital.

Sir,—Referring to your recent article on the hardship due to the closing of all chemists' shops one afternoon in the week, and Mr. G. P. Wyatt's (the Coroner) remark that "possibly those who made the regulations were people who knew nothing about it," I may point out that chemists are exempt from the provisions of the Shops Act. They have, however, in all parts, like sensible men, voted themselves in the Act, and thus secured a half-holiday for themselves, which they badly need.

There is not a body of men on the face of the earth who work longer hours than chemists, and the half-holiday has been a perfect godsend to them. Doctors all know the early-closing day for their district, and it should be quite easy for them to supply their patients in extreme cases with medicine on the early-closing day.—Faithfully yours,

Albert Larking, Secretary.

Early Closing Association, 3 Tudor Street, E.C.

